# The Ophthalmo-Tuberculin Reaction

**Published:** 1909-05

**Authors:** Herman D. Andrews


### The Ophthalmo-tuberculin Reaction

[Abstracted by HERMAN D. ANDREWS, M. D.]

A REPORT of 1,087 instillations of tuberculin in the conjunctival sac by a uniform method was made by Dr. E. R. Baldwin in the issue of the Journal of the American Medical

Association, February 20, 1909. He obtained uniformity of procedure by furnishing all the different observers the same preparation of old tuberculin (human) in the strength of 0.3 and 0.5 per cent. and including pipettes graduated to 0.25 cubic centimeter. In all, 887 individuals were tested, in 190 the test being repeated. There were 310 cases of tuberculosis in some form, 265 suspected of tuberculosis, 127 subject to other disease and 185 supposedly healthy subjects.

In the records he found that in incipient stages of tuberculosis 67 per cent. of the tests were positive; in the moderately advanced 75 per cent. were positive, while in far advanced cases, the proportion dropped to 50 per cent. As an average for all the cases known to be tubercular 70 per cent. of the tests were positive. Of the cases of suspected pulmonary tuberculosis only 33 per cent. reacted, while in suspected other forms of tuberculosis 48 per cent. were positive. Reactions in other diseases were but 14 per cent. while healthy subjects showed 18 per cent. positive. In the incipient cases, where we wish most to be aided in diagnosis 71 per cent. were positive, but in the suspected cases where we could expect assistance in diagnosis only 33 per cent. were positive. As an ideal diagnostic method in tuberculosis the results show that the test falls short of the requirements, though relatively good in confirmation of diagnosis in clinically tuberculous cases.

The significance of age as a factor influencing the reaction cannot as yet be determined, but in this series, tuberculous cases between ten and fifteen years gave the highest percentage of positive reactions. Fever had apparently no influence on the chance of reaction.

Control tests were made with the cutaneous and subcutaneous methods. Results in both of these tests were positive more frequently than in the ophthalmo-test. As possible reasons accounting for the nonreaction of the latter may be named the following: uncertain absorption of the tuberculin; smallness of the dose required for safety; variations of the susceptibilities to the reaction in different individuals.

As to the clinical value of the ophthalmo-tuberculin reaction for prognosis clinicians are being impressed with its limitations. A positive reaction cannot be interpreted as an infallible criterion of existing disease. It only betrays the presence of infection, and whether it is recent or long healed must be determined by other means. In reality, reaction which takes place only to large or repeated doses of tuberculin are of little clinical value considered apart from other symptoms. The absence of reaction after such application is of far greater value in excluding tuberculosis. From his series, the writer feels that the advantages of the conjunctival test are found in cases where positive results follow the use of very small amounts of tuberculin and that only absence of reaction to repeated and large doses has any value in excluding tuberculosis.

As to the danger locally, the writer found severe conjunctivitis in ten cases and one case of keratitis in a patient which had tubercular lesions, but in general, there are no great risks in the use of the non-repeated weak solution. However, the ill-results that have been reported should lead one to make the test with a feeling of responsibility.

Among other conclusions the writer states that: the conjunctival-tuberculin test performed with a single instillation has some value in confirming the presence of tuberculosis in the early stages; it is of little value in confirmation when the symptoms of tuberculosis are only suspicious; the test is unreliable for prognosis; with proper precautions, danger to the eye is slight and need not preclude the test when other methods are inapplicable, as when fever is present; its use should be restricted to adults.

